# The Role of Fibrosis in Osteoarthritis Progression

**DOI:** 10.3390/life11010003

**Published:** 2020-12-23

**Authors:** Yeri Alice Rim, Ji Hyeon Ju

**Affiliations:** 1 Catholic iPSC Research Center, College of Medicine, The Catholic University of Korea, Seoul 06591, Korea; llyerill0114@catholic.ac.kr; 2 Division of Rheumatology, Department of Internal Medicine, Seoul St. Mary’s Hospital, Institute of Medical Science, College of Medicine, The Catholic University of Korea, Seoul 06591, Korea

**Keywords:** osteoarthritis, articular cartilage, chondrocyte, synovium, synoviocyte, fibrosis

## Abstract

Osteoarthritis (OA) is a chronic degenerative joint disease where the main characteristics include cartilage degeneration and synovial membrane inflammation. These changes in the knee joint eventually dampen the function of the joint and restrict joint movement, which eventually leads to a stage where total joint replacement is the only treatment option. While much is still unknown about the pathogenesis and progression mechanism of OA, joint fibrosis can be a critical issue for better understanding this disease. Synovial fibrosis and the generation of fibrocartilage are the two main fibrosis-related characteristics that can be found in OA. However, these two processes remain mostly misunderstood. In this review, we focus on the fibrosis process in OA, especially in the cartilage and the synovium tissue, which are the main tissues involved in OA.

## 1. Introduction

Osteoarthritis (OA) is a common joint disease where the primary risk factors include traumatic joint injury or the mechanical disruption of joint tissues due to accumulated external forces. The etiology is multifactorial, and both environmental and genetic factors are thought to contribute to triggering the pathogenesis and progression of OA [1]. Critical hallmarks of OA include cartilage degeneration, osteophyte formation, and fibrosis in the joint tissue [2]. When left untreated, the defected joint eventually requires total joint replacement.

Tissue fibrosis occurs in various tissues, including the liver, kidney, heart, and others, and it eventually leads to organ failure. Fibrosis also occurs in the articular joint during the progression of OA and plays a critical role in OA pathogenesis and progression as well as cartilage destruction [3]. Joint fibrosis is characterized by the excessive accumulation of connective tissue, which eventually contributes to joint stiffness that results in intense pain during joint movement [4]. While the articular cartilage is unable to repair itself, affected chondrocytes often undergo de-differentiation and convert into fibrotic chondrocytes. Then, fibrotic chondrocytes secrete proteins that are similar to fibrocartilage, which is stiffer and mechanically inferior compared to the original hyaline cartilage [5,6]. Then, the cartilage defect recovers with fibrocartilage-like tissue that lacks its original function and even worsens the symptoms of OA [7]. This process also occurs in the cells (e.g., primary chondrocytes, mesenchymal stem cells) that are applied for OA therapy [8]. Synovitis caused by hyperplasia in the synovial membrane is also thought to be related to fibrosis [9].

In the past, OA was considered a disease that affects cartilage only; however, it is now recognized as a more complex disease. Not only is cartilage involved, but the subchondral bone, ligaments, and the synovium are all orchestrated under an inflamed environment, and fibrosis is thought to be included in this process [3]. In this review, the impact of fibrosis in the pathogenesis and progression of OA will be discussed.

## 2. Articular Cartilage and OA

Articular cartilage in the knee joint usually refers to hyaline cartilage, which is a lubricated smooth surface that absorbs the external forces to protect the ends of bones [10]. While chondrocytes are quiescent cells with a low proliferation rate, they have a highly active protein-synthesizing ability [11]. Articular cartilage is mainly composed of extracellular matrix (ECM) secreted by embedded chondrocytes, and only a small percentage of chondrocytes exist within the cartilage tissue [12]. Due to this unique composition, no blood vessels, lymph nodes, or nerves can be found in this tissue, which makes it more difficult to recover after damage because the delivery of nutrition or stem cells to the affected area is blocked by the thick ECM between each cell [10]. Articular cartilage has a poor intrinsic capability for self-repair [13].

OA is a common joint disease that is characterized by deformity and pain, which leads to a reduction in joint motion and function [14,15]. The main characteristic of OA is the defected or degraded cartilage tissue [15]. Defects in the cartilage tissue induced by trauma or aging can trigger the pathogenesis and progression of OA [16]. While OA can occur in relatively younger populations as a result of focal defects caused by extreme movements through activities such as sports, it usually affects the elderly population due to excessive use of the joints over time [17]. Classically, chondrocytes are categorized by the types of ECM proteins that they express [18]. Healthy articular chondrocytes that exist within the healthy articular cartilage are reported to express high levels of aggrecan and collagen type II, which are critical markers for hyaline cartilage [19]. However, the de-differentiation of chondrocytes in cartilage lesions induce the production of a reduced quality of hyaline cartilage, leading to the increased expression of fibrotic or OA markers such as collagen type I [20].

Repair of the articular cartilage has been attempted for several decades with various strategies, including cell-based therapies [15,21,22]. In previous studies, primary chondrocytes or adult stem cells such as mesenchymal stem cells (MSCs) have been widely used for the treatment of cartilage lesions [5,23,24]. However, even with improved outcomes, one of the main disadvantages of cartilage regeneration or repair is the generation of fibrotic cartilage instead of hyaline cartilage [8]. The implanted or therapeutically delivered cells in the cartilage defect undergo fibrosis and induce fibrotic cartilage formation with an increased expression of collagen type I [8].

While fibrocartilage and fibrogenic chondrocytes can be found in other cartilaginous tissues such as the menisci or tendon, they have been shown to be detrimental when found in the articular cartilage [12]. Blocking the development of fibrocartilage and fibrosis remains a hurdle for cartilage treatment and regeneration.

## 3. Synovium and OA

Synovium, also referred to as the synovial membrane, lines the inner surface of the joint tissue (except for the articular cartilage) [25]. The major role of synovial tissue is to seal the synovial cavity and fluid to prevent leakage to other tissues. The synovium produces synovial fluid, which offers nutrition and oxygen to chondrocytes embedded in the articular cartilage by diffusion [26]. It is also responsible for synovial fluid maintenance by controlling the composition and total volume. Synovial fluid maintains the lubricated environment in the joint cavity by secreting lubricin and hyaluronic acid, which protect the cartilage from wear and tear [3].

Normal synovium is composed of two layers. The outer layer (subintima) is about 5 mm thick and consists of various connective tissues [27]. This layer has a large proportion of collagen type I. The inner layer (intima) directly lines the joint cavity, with a thickness of only 20–40 μm. The synovial membrane in OA joints is characterized by hyperplasia, vascularization, immune cell infiltration, increased levels of inflammatory molecules, and fibrosis [28]. The thickness of the synovium is significantly increased in OA. Several studies have suggested that synovitis is related to pain, and it can be a possible candidate for an OA progression trigger [28]. Histological changes in the OA-affected synovium include hyperplasia in the synovial lining and sub-lining fibrosis. Unlike the articular cartilage, the synovium contains abundant blood and nerve supply throughout the tissue [9]. As a result of this composition, the synovium is the main location where the infiltration of inflammatory cells occurs, which induces inflammation in the OA knee joint. Angiogenesis also contributes to chronic synovitis, which is thought to increase the inflammation in the OA knee joint [29]. In later stages of OA, surface fibrin deposition and fibrosis within the synovium can be commonly observed [30].

Recently, it has been suggested that the infrapatellar fat pad (IFP) and synovial membrane are one anatomofunctional unit rather than being recognized as two separate tissues [31,32]. The IFP, also known as Hoffa’s fat pad, is located below the knee cap (patella), specifically between the joint capsule and the synovial membrane [31]. It has several roles in the knee joint. The IFP protects the joint from mechanical stress by stabilizing the patella during exercise [33]. It also provides a vascular supply to the patellar tendon and the interior patellar pole [34]. The main cell type in the IFP is adipocytes; however, fibroblasts and other immune cells are also found [35]. Similar to other adipose tissues, the IFP secretes various cytokines and adipokines [36], and the IFP is reported to contribute to OA pathogenesis and the development of synovial fibrosis by promoting cell proliferation and migration [37]. The IFP is also suggested as the cause of anterior knee pain [38]. The IFP also interacts with other periarticular tissues, including the articular cartilage and the synovial membrane, during the progression of OA [39]. While synovitis and synovial fibrosis are thought to be critical to control OA, much is still unknown about this process.

## 4. Fibrosis and OA

Fibrosis is usually a wound healing process [3]. The classic wound healing process results in cell proliferation, differentiation, ECM production, and remodeling. However, an abnormal wound healing process can lead to the excessive secretion and deposition of ECM proteins in the tissue, followed by excessive fibrosis, which eventually results in scar formation, inflammation, and even damage in the tissue [40].

**Figure 1:**
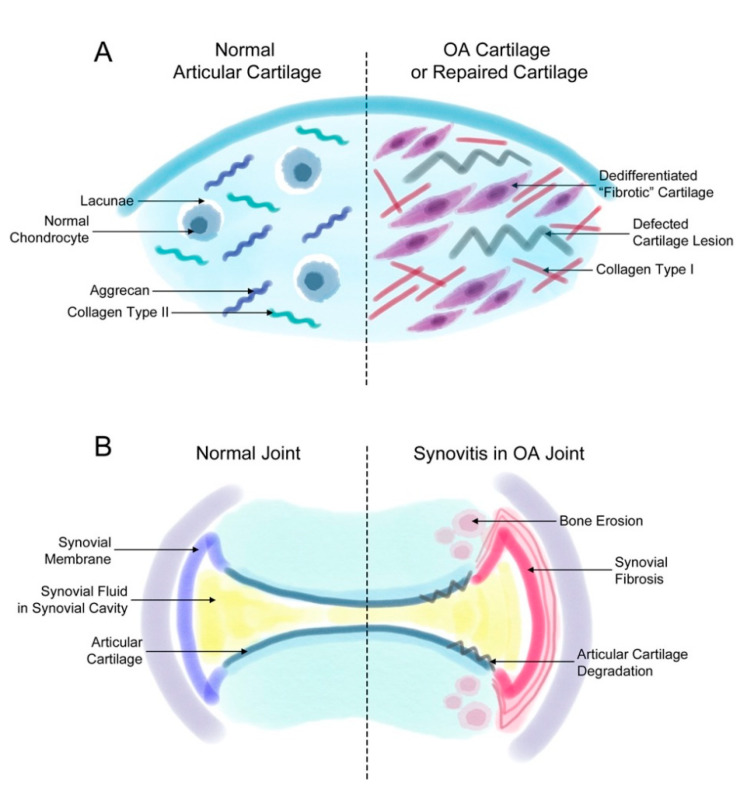
Fibrosis in synovial and cartilage tissue. (**A**) The normal joint consists of a smooth layer of articular cartilage and a smooth layer of synovial membrane on the side that maintains the synovial fluid in the synovial cavity. In the joint of a patient with osteoarthritis (OA), the increased proliferation of synovial cells induces synovial fibrosis that results in joint swelling, stiffness, and pain. The increasing levels of synovium eventually affect the cartilage and bone tissue, inducing further degradation of the cartilage tissue and bone erosion. (**B**) The normal articular cartilage has a smooth extracellular matrix (ECM) that is mostly composed of aggrecan and collagen type II. Normally, chondrocytes remain in small spaces called lacunae. However, chondrocytes in a defected cartilage lesion undergo abnormal proliferation that leads to their de-differentiation into fibroblast-like fibrotic chondrocytes. Then, these chondrocytes secrete ECM proteins such as collagen type I instead of aggrecan or collagen type II. These changes lead to a stiffer type of cartilage and eventually completely change the characteristics of the articular cartilage.

**Figure 2:**
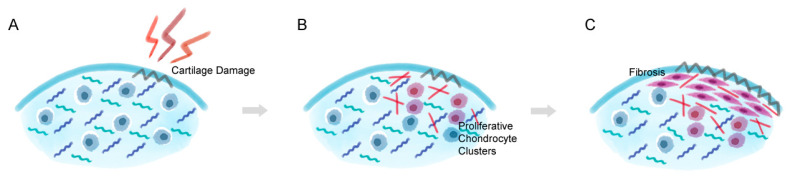
Chondrocyte de-differentiation process in cartilage lesion. (**A**) When cartilage damage occurs, (**B**) chondrocytes that are embedded near the lesion become actively proliferative and start to change the surrounding environment by synthesizing collagen type I. (**C**) The proliferative chondrocytes eventually alter into a fibroblast-like characteristics and form a thick layer of fibrocartilage-like tissue and induce degradation and dedifferentiation in the nearby hyaline cartilage tissue.

Fibrosis that occurs during OA induces joint stiffness and pain. As mentioned earlier, fibrosis is found in two events related to OA: (1) the generation of fibrotic cartilage during cartilage repair or OA progression (Figure 1A) and (2) synovial fibrosis during the onset and progression of OA (Figure 1B).

### 4.1. De-Differentiated Chondrocytes in OA

The composition of secreted ECM proteins by chondrocytes is altered in response to environmental changes within the joint, and the quality of cartilage is maintained by a balance of catabolic and anabolic processes [41]. The imbalance between these two processes under a diseased environment results in cartilage loss and degeneration. In the early stages of OA, quiescent articular chondrocytes become actively proliferative and form cell clusters in the cartilage lesion to adjust to changes in the cartilage microenvironment (Figure 2) [42,43]. The proliferative chondrocytes transit or de-differentiate into a fibroblastic phenotype that undergoes a drastic change in cell shape, metabolism, and cytoskeletal structure, eventually leading to the increased synthesis of collagen type I, which weakens the cartilage tissue by forming a mechanically-incompetent repaired fibrocartilage-like tissue [44]. The reduction in collagen type II expression and the increased expression of collagen type I is the main characteristics of articular chondrocyte de-differentiation and fibrosis [45]. In normal articular hyaline cartilage, only 1.7% of the total area is composed of collagen type I, while collagen type II makes up almost 100% [46]. Biochemical analysis also shows that 0.2% of the total collagen in the articular cartilage is collagen type I, and 96% is collagen type II [47]. Unlike hyaline cartilage, fibrocartilage has a low percentage of aggrecan and collagen type II, is rich in collagen type I, and has limited durability [48,49]. Despite the synthetic activity that de-differentiated chondrocytes maintain, the expression of aggrecan and collagen type II ceases and the secretion of collagen type I begins [50]. Isolated chondrocytes also undergo de-differentiation, and in vitro de-differentiated chondrocytes also express OA-related proteins, lose the hyaline chondrocyte phenotype, and gain a fibroblast-like phenotype [49,51,52]. After this proliferation process in advanced stages of OA, chondrocytes within the affected cartilage undergo de-differentiation and obtain a fibroblast-like phenotype that produces abnormal components such as fibronectin fragments and induce the formation of fibrocartilage instead of hyaline cartilage [51,53]. These changes in the cartilage environment ultimately reset the chondrocyte cell cycle and lead to abnormal chondrocyte proliferation and hypertrophic differentiation, which eventually results in chondrocyte apoptosis [50,51]. While the hypertrophic differentiation of chondrocytes is a normal pathway in developmental events, abnormal hypertrophic differentiation in adult cartilage is one of the causes of OA [54].

### 4.2. Synovial Fibrosis in OA

**Figure 3:**
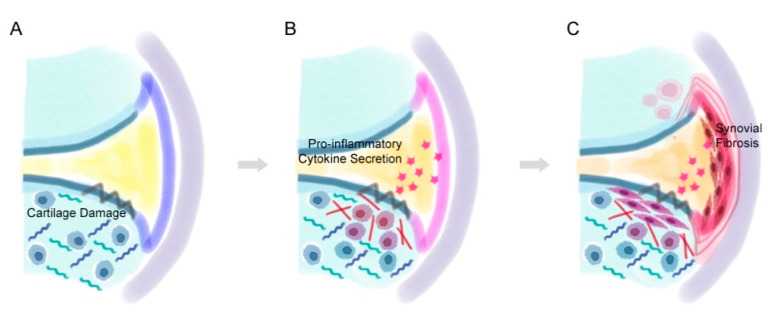
The process of synovial fibrosis. (**A**) After cartilage damage occurs, (**B**) the abnormally proliferative chondrocytes near the lesion secrete pro-inflammatory factors that inflames the synovial membrane. (**C**) The pro-inflammatory factors eventually lead to hyperplasia of the synoviocytes and thicken the lining of the synovial membrane.

While synovitis occurs in various joint diseases, it is also a common symptom that is commonly found in OA [28]. Synovitis is a broad term for inflammation in the synovial membrane and is common in both early and late OA [9,55]. Synovitis in OA patients can be characterized by hyperplasia (thickening) of the cells lining the synovial membrane, increased stromal vascularization, the infiltration of inflammatory cells, and fibrosis of the sub-lining synovial cells (Figure 3) [28]. Synovial fibrosis is one of the characteristics of synovitis and also occurs also during the progression of OA. It is characterized by excessive fibroblast proliferation and an imbalance between collagen synthesis and catabolism [25,56]. In patients with OA, synovial tissue affected by fibrosis becomes thicker and rigid [30]. These symptoms contribute to the main characteristics of OA, such as joint pain and stiffness [57]. Fibrosis results in fibrin deposition in the synovial tissue [58]. While inflammation is observed more in the early stages of OA, it is reported that fibrosis was observed more in the late stages of OA [3,59].

While cartilage damage or degradation is the main characteristic of OA, molecules secreted from the degraded articular cartilage that are released into the synovial cavity initiate synovial inflammation [28]. Synoviocytes in the synovial membrane react to this process by producing pro-inflammatory cytokines and growth factors that attract immune cells by increasing angiogenesis and inducing both a phenotypic shift of articular chondrocytes and synovial fibrosis [60]. The affected de-differentiated articular chondrocytes then produce additional inflammatory cytokines and proteolytic enzymes that induce cartilage degradation and synovial inflammation [28,61]. Transforming growth factor beta (TGFβ)-related pathways are thought to be the most critical type of pathway involved in the onset of synovial fibrosis. While TGFβ usually works as a positive mediator that promotes ECM protein synthesis in the cartilage, it can also cause fibrosis. TGFβ induces synovial fibrosis, attracts immune cells such as leukocytes, and induces osteophyte formation. The injection or overexpression of TGFβ has induced fibrosis in animal models of OA [62,63].

Angiogenesis and fibrosis are closely related to one another, and blood supply seems to be crucial during tissue fibrosis [64]. Various studies confirmed fibrosis in OA IFP [32,65,66]. The IFP forms numerous blood vessels with fibrous tissues, including the synovial membrane [67]. The formation of blood vessels in the IFP is reported to heal lesions that occur near the anterior cruciate ligament and other nearby tissues; however, this process also hastens the development of fibrosis in the knee joint after injury [34]. OA IFP has higher levels of vascular endothelial growth factor (VEGF) and is associated with the increased formation of blood vessels, along with the higher expression of monocyte chemotactic protein 1 (MCP-1) and interleukin (IL)-6 [68]. Higher expression of IL-6 in OA IFP was associated with increased levels of synovial membrane fibrosis, which proves that OA IFP affects the fibrosis process in the synovial tissue [66].

While OA IFP affects fibrosis in the synovial membrane, it has been also shown that the fibrotic environment also affects the OA IFP tissue in reverse. Inter-alpha-trypsin inhibitors (ITIs) are plasma protease inhibitors that contributes to ECM stability by forming complexes with covalent linkage to hyaluronan [69]. While inter-alpha-trypsin inhibitor heavy chain 5 (ITIH5) was reported to be highly expressed in white adipose tissue and obese subjects, Belluzzi et al. confirmed the increased expression of ITIH5 after observing increased fibrosis in the in OA IFP [70]. The study showed evidence for the first time that suggest the relationship between OA pathology and the expression of collagens and adipokines involved in fibrosis. This study also suggests the OA IFP tissue, especially its relationship with tissue fibrosis, as a possible therapeutic target to prevent OA progression.

## 5. Fibrosis-Related Markers in OA

Two main factors that are critical for fibrotic disease, namely TGFβ and connective tissue growth factor (CTGF), are also elevated in OA and are the main candidates for the development of joint fibrosis. The overexpression of CTGF in the joints of mice induced synovial fibrosis [63]. CTGF expression can be induced by TGFβ; however, it can also function independently [2,3,71]. Scharstuhl et al. showed that TGFβ plays an important role in synovial thickening by fibrosis in experimental OA, and also that blocking this pathway prevented this process [72]. TGFβ promotes the formation of ECM by inducing collagen and fibronectin synthesis [73]. TGFβ also plays a critical role in fibrosis in various organs and is known to control cell proliferation, differentiation, immunity, and wound healing [2]. While TGFβ plays a critical role in cartilage fibrosis in patients with OA, blocking this molecule is not a treatment option since it is also a critical factor necessary for chondrogenesis. High levels of TGFβ exist in healthy cartilage, while low levels are found in OA cartilage [74]. Adequate levels of TGFβ even had protective effects on cartilage in animal models of arthritis; however, an excessive amount of this growth factor had adverse effects [75]. It is also reported that high concentrations of TGFβ exist in OA synovial fluids, which is produced by synoviocytes [76]. The administration of 20 ng of TGFβ was enough to increase the number of synovial lining cells by inducing fibroblast proliferation, along with collagen deposition [77]. The delivery of TGFβ by injection or transfection also resulted in increased synovial hyperplasia and osteophyte formation [78]. High levels of TGFβ activate the SMAD1-5-8 pathway instead of the SMAD2-3 pathway. The activation of the SMAD1-5-8 pathway upregulates genes related to fibrogenic differentiation and hypertrophy, and eventually induces synovial fibrosis and osteophyte formation [79]. However, it is reported that the difference between the two major growth factors is that fibrosis induced by TGFβ is relatively persistent, while CTGF-induced fibrosis is relatively temporary (about 28 days) [2]. Moreover, the viral delivery of TGFβ increased COL1A1 expression, but CTGF overexpression did not [80].

Collagen type I is also a well-known marker for fibrosis and has been detected in various fibrosis-related diseases, including OA [81,82,83]. Collagen type I and alpha-smooth muscle actin (α-SMA) are key markers of both fibrocartilage formation and synovial fibrosis [84]. Remodeled cartilage in the defects of osteoarthritic cartilage show fibrotic characteristics such as increased levels of collagen type I and α-SMA [85,86,87]. Collagen type I is a major marker of unfavorable fibrocartilage and chondrocyte de-differentiation [8,88]. As mentioned earlier, in vitro de-differentiated chondrocytes express the OA-related de-differentiation marker collagen type I. Articular chondrocytes that undergo longer expansion times with multiple passages in monolayer culture end up in chondrocyte de-differentiation [89]. During the de-differentiation process, chondrocytes go through morphological and phenotypical changes and become fibroblastic, eventually producing collagen type I [88]. The compositional changes in the ECM also affect the chondrogenicity of MSCs [43]. MSCs cultured in collagen type II hydrogels showed higher expression of chondrogenic markers, which might indicate chondrocyte de-differentiation caused by collagen type I [90]. During OA progression, residential chondrocytes producing collagen type I might affect the subsequent chondrogenesis of MSCs [91]. Collagen type I is the major marker for fibrotic de-differentiated hyaline cartilage, but collagen type III is also thought to be related to the de-differentiation process, which suggests that it could also be a marker for the fibroblast-like phenotype. TGFβ1 markedly increased the SMA content in chondrocytes [92]. The secretion of α-SMA by OA chondrocytes has been verified and was observed to significantly increase in OA cartilage, along with collagen type III [51,84].

The de-differentiated chondrocytes and fibrocartilage formed in the articular cartilage lesion produce pro-inflammatory mediators that further induce cartilage degeneration and synovial inflammation. IL-1β has been identified as the most prevalent cytokine in OA, which is involved in cell proliferation, differentiation, and apoptosis [93]. IL-1β also interferes with the production of hyaline cartilage structural proteins, collagen type II, and aggrecan by altering chondrocyte characteristics.

Proteases are actively involved in the pathogenesis of OA. Proteases, including matrix metallopeptidases (MMPs), disintegrin and metalloproteinase with thrombospondin motifs (ADAMTSs), mainly degrade the main hyaline cartilage ECM proteins aggrecan and collagen type II [94]. MMPs are a zinc-dependent enzyme family that degrades ECM proteins in the articular cartilage. Various types of MMPs exist and can be categorized into several groups: collagenases, gelatinases, stromelysins, metalloelastases, and more. Of all the MMPs, MMP13 is considered to be the most important agent for collagen degradation that leads to OA, as it degrades collagen type II [95,96]. Some MMPs have also been proposed to regulate ADAMTS activity [97]. ADAMTSs eventually circulate in a vital cycle between the degenerated chondrocyte and other cells near it, such as synoviocytes in the synovial membrane. These proteases are also reported to be related to fibrosis in several diseases including idiopathic pulmonary fibrosis, liver fibrosis, and others [98,99]. Therefore, they are also thought to be related to joint fibrosis; however, there is currently little information on the direct relationship between the two factors.

Other markers such as procollagen lysine, 2-oxoglutarate 5-dioxygenase 2 (PLOD2), and tissue inhibitor of metalloproteinase 1 (TIMP1) are reported to increase in OA-related fibrosis and are also considered fibrotic markers [3,100]. PLOD2 is an enzyme related to collagen crosslinking that induces pyrodinoline crosslink formation [3]. Pyrodinoline crosslinks make collagen fibers resistant to collagen degradation, which makes them more rigid and results in collagen accumulation in fibrotic tissues [101]. Collagen accumulation is induced by reduced levels of collagen degradation, inducing increased pyridinoline crosslinks per collagen triple helix [101]. Remst et al. confirmed the presence of increased levels of PLOD2 and an increased pyridinoline crosslink/collagen ratio in OA synovium, and this expression increased along with the severity of OA. While the presence of fibrosis in OA patients was unknown, it is expected that PLOD2 may have higher expression in the subpopulation of OA patients with fibrosis. Ueki et al. confirmed that the knockout of PLOD2 inhibited the tumorigenesis role of integrin β1 in tumor cells [102]. While it is still controversial, integrins are reported to be closely related to the pathogenesis of OA, and the confirmation of this process in OA might suggest a new candidate for OA treatment [103,104]. TIMP1 is an inhibitor of matrix metallopeptidases (MMPs), which are well known for their role in OA. The elevation of TIMP1 expression was confirmed in various fibrotic diseases, including synovial fibrosis in OA [105]. TIMP1 elevation in fibrotic tissue is induced by elevated levels of TGFβ, and TIMP1 induces fibrosis via the downregulation of MMPs [106]. The relationship between hypoxia and synovial fibrosis has been also shown by observing the expression of HIF-1α; the expression of TGFβ, COL1A1, PLOD2, and TIMP1 was downregulated by the inhibition of HIF-1α in rat models of OA [107].

## 6. Strategies for OA Treatment Targeting Fibrosis

Various studies have shown evidence of a scarring response in OA joint tissues [41,108,109,110,111]. These studies suggest that fibrotic scarring of the joint tissue can be an important target for OA therapy.

Liu and colleagues reported that asiatic acid (AA) reduces the hypertrophic and fibrotic differentiation of chondrocytes [84]. AA is a pentacyclic triterpene that exhibits various clinical effects, including anti-oxidant and anti-inflammatory effects. While AA already has already shown positive effects in liver fibrosis, it has also successfully reduced the levels of collagen type I and α-SMA in OA models. This process was achieved by regulating the AMP-activated protein kinase (AMPK)/phosphoinositide 3-kinase (PI3K)/protein kinase B (Akt) signaling pathway.

TGFβ and activin receptor-like kinase 5 (ALK5) is thought to be a critical cause of synovial fibrosis [3]. Blocking ALK5 promoted MMP13 expression and reduced the expression of collagen type II in chondrocytes [112]. While the TGFβ/smad signaling pathway is already known to be closely related to fibrosis in various tissues, the increased expression of smad2/3 and smad4 was observed in the scar tissue prevalent after myocardial infarction [113]. Based on this result, Xue et al. confirmed the role of smad4 in fibrosis by knocking down the smad4 gene in rat synovial cells and chondrocytes [73]. The knockdown of the smad4 gene decreased the proliferation rate of cells induced by TGFβ1, along with the expression of other fibrosis markers such as vimentin, α-SMA, collagen type I, and TIMP1.

Fibrosis during cartilage de-differentiation is a critical issue in in vitro chondrogenesis for the development of regenerative medicine for defected joints, particularly for the culture of cartilage tissue. The increased expression of collagen type I and type X is a major issue in in vitro cartilage regeneration and in repaired cartilage by transplanted cells [13,114]. While the expansion of primary chondrocytes readily results in de-differentiation, research on the generation of chondrocytes from progenitor cells is ongoing. While adult stem cells such as MSCs have mainly been used prior to the isolation of pluripotent stem cells, currently, in vitro chondrogenesis is actively done using induced pluripotent stem cells (iPSCs). Using several growth factors such as TGFs or bone morphogenetic proteins (BMPs), in vitro chondrogenesis has been attempted using various methods. The three-dimensional culture of stem-cell-derived chondrogenic progenitor cells showed the highest differentiation efficacy and a lower expression of fibrotic markers [13,114,115,116]. Despite several decades of progress in this area of research, the generation of a stable chondrocyte using progenitor cells or stem cells remains a major hurdle for cartilage regeneration and treatment [117].

## 7. Conclusions

The initiation and progression of OA is a complex process. The reduction in fibrotic markers such as collagen type I should be a major focus of OA treatment. In this review, we focused on the two fibrosis processes that are related to the key characteristics of OA: (1) fibrosis induced by chondrocyte de-differentiation and (2) synovial fibrosis in synovitis. Much remains unknown about the relationship between OA and fibrosis. Therefore, further study on fibrosis in OA should be conducted to gain a better understanding of this potential therapeutic target for OA treatment.
